# Low BMI, blood calcium and vitamin D, kyphosis time, and outdoor activity time are independent risk factors for osteoporosis in postmenopausal women

**DOI:** 10.3389/fendo.2023.1154927

**Published:** 2023-10-23

**Authors:** Guo Tang, Long Feng, Yu Pei, Zhaoyan Gu, Tingting Chen, Zeguo Feng

**Affiliations:** ^1^ Department of Pain, The First Medical Center of People's Liberation Army (PLA) General Hospital, Beijing, China; ^2^ Department of Anesthesiology, People's Liberation Army (PLA) General Hospital of Hainan Hospital, Sanya, Hainan, China; ^3^ Department of Endocrinology, The First Medical Center of People's Liberation Army (PLA) General Hospital, Beijing, China; ^4^ Department of Endocrinology, The Second Medical Center of People's Liberation Army (PLA) General Hospital, Beijing, China; ^5^ Department of Anesthesia and Cardiopulmonary Bypass, Cardiovascular Medical Department, The Sixth Medical Center of Chinese People’s Liberation Army General Hospital, Beijing, China

**Keywords:** blood calcium, blood vitamin D, females, kyphosis, outdoor activity time, postmenopausal osteoporosis, osteoporosis

## Abstract

**Aim:**

To explore the risk factors of osteoporosis in postmenopausal women in China.

**Method:**

This study collected all patient data from January 2014 to December 2015. Basic information and questionnaires were collected from 524 postmenopausal women in Sanya and Hainan Province. The questionnaire was administered to the enrolled participants by endocrinologists. Biochemical parameters were measured using fasting blood samples, and bone density was measured by dual energy X-ray absorptiometry at the department of radiology of Hainan hospital, PLA General Hospital. Participants with an R-value of ≤-2.5 were diagnosed with osteoporosis. After deleting missing values for each factor, 334 participants were divided into the osteoporosis (n=35) and non-osteoporosis (n=299) groups according to the R-values.

**Results:**

The participants had a median age of 60.8 years (range: 44–94 years). Among the 334 postmenopausal women included in this study, 35 (10.5%) were diagnosed with osteoporosis. Univariate analysis showed statistically significant differences in age, BMI, type of work, alkaline phosphatase, years of smoking, blood calcium levels, kyphosis, fracture, and asthma between the two groups (P<0.05). In addition, multivariate logistic analysis showed that age (odds ratio [OR]: 1.185, 95% confidence interval [CI]: 1.085–1.293, P<0.001) and kyphosis times (OR:1.468, 95% CI: 1.076–2.001, P=0.015) were positively correlated with postmenopausal osteoporosis, whereas BMI (OR: 0.717, 95% CI: 0.617–0.832, P<0.001), blood calcium levels (OR: 0.920, 95% CI: 0.854-0.991, P=0.027), vitamin D levels (OR: 0.787, 95% CI: 0.674–0.918, P=0.002), and outdoor activity time (OR: 0.556, 95% CI: 0.338-0.915, P=0.021) were negatively correlated with postmenopausal osteoporosis.

**Conclusion:**

Low BMI, blood calcium and vitamin D levels, kyphosis time, and outdoor activity time are independent risk factors for osteoporosis in postmenopausal women.

## Introduction

Osteoporosis (OP) is a systemic skeletal disorder characterized by low bone mineral density (BMD), loss of bone mass, microarchitectural deterioration, and loss of bone quality. Up to 71% of osteoporotic fractures occur in women aged 50 years and older (1, 2). In the United States, half of the women older than 50 years have osteoporotic fractures (3). Older postmenopausal women are prone to OP, which increases the risk of fractures associated with reduced quality of life, disability, economic burden, morbidity, and mortality (4–6). It is associated with estrogen deficiency, advanced age, heredity, smoking, leanness, and several diseases and drugs that impair bone health (7). In the United States, the cost of OP-related fractures was $17 billion in 2005, and the cost is expected to increase to $25.3 billion by 2025 (8). OP is also a significant disease in China; OP fractures require long-term hospitalization, and Chinese patients have the highest cost of all fractures, with an estimated 2.33 million OP-related fractures occurring in 2010 and a treatment cost of $9.45 billion, which is expected to rise to 5.99 million fractures at a cost of $25.43 billion USD by 2050 in China (2).

Postmenopausal osteoporosis (PMOP) is an asymptomatic skeletal disease that is often underdiagnosed and undertreated. Furthermore, the increasing burden of socioeconomic medical care and families with OP fractures worldwide highlights the needs to continue to improve the diagnosis of OP and to identify the risk factors for OP (7, 9). This was a cross-sectional study conducted in Hainan Province, Sanya City, China, to explore the risk factors for developing osteoporosis in postmenopausal women.

## Methods

### Study population

**Figure 1:**
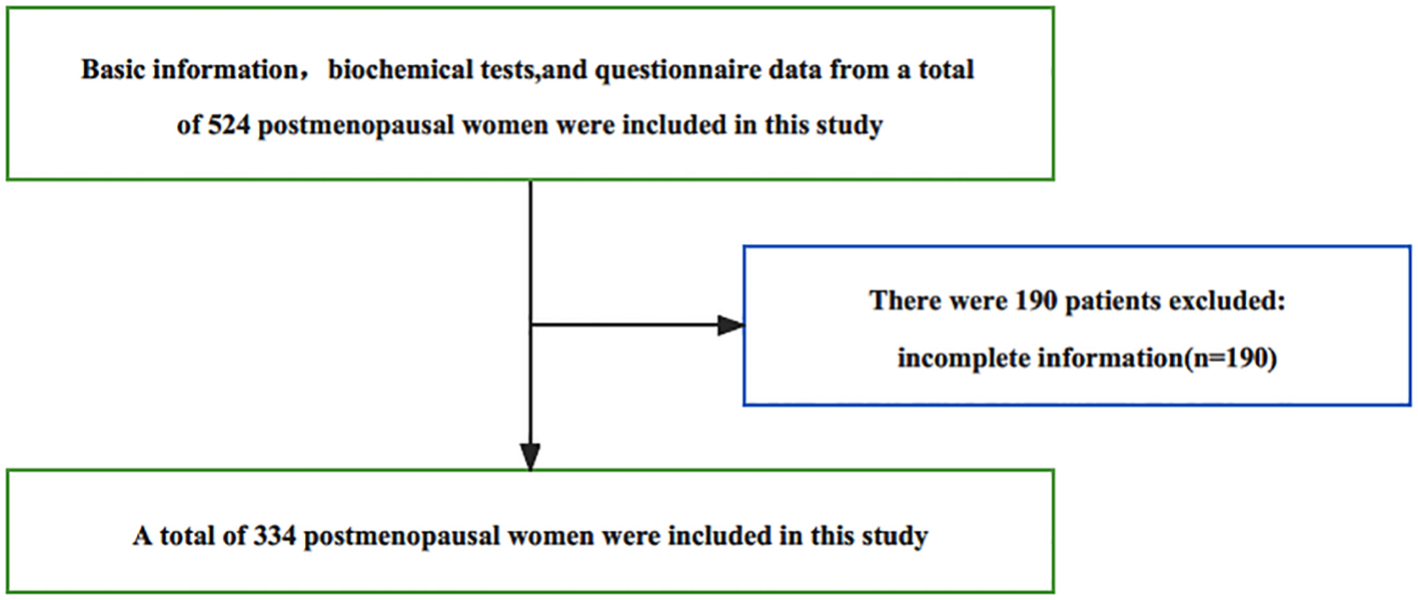
Numbers of centenarians included and excluded in this study.

This study collected all data from January 2014 to December 2015. Basic information and questionnaires were collected from 524 postmenopausal women in Sanya and Hainan provinces. After deleting the missing values for each factor, 334 participants were divided into osteoporosis (n=35) and non-osteoporosis (n=299) groups according to the R-values (**Figure 1**). The questionnaire was administered to the enrolled participants by endocrinologists. In addition, the same caregiver was assigned to collect the biochemical parameters of each participant by going to the patient’s home to draw fasting blood, which was then sent to the Department of Biochemistry, Hainan hospital, PLA General Hospital for examination. Bone density was measured at the department of radiology of our hospital.

### Definitions and diagnostic and exclusion criteria for postmenopausal OP

To evaluate whether postmenopausal OP was present, this study used the R-value determined by dual-energy X-ray absorptiometry (DXA) in all postmenopausal women. BMD measured using DXA is currently considered the gold standard for diagnosing OP (10). According to the World Health Organization (WHO) criteria, OP is defined as a low BMD (> 2.5 SD below the mean) at any site in the lumbar spine (L1-L4), femoral neck, or total hip. It has been suggested that the diagnosis of OP can be based on fractures without significant trauma or low BMD, as measured using DXA. Furthermore, an R-value of ≤ -2.5 is sufficient for the diagnosis of OP (11). The exclusion criteria for this study included the use of OP drugs (such as bisphosphonates and teriparatide), corticosteroid drugs, anorexia nervosa, Cushing’s syndrome, hyperparathyroidism, and kidney disease. In addition, possible causes of secondary OP, such as osteogenesis imperfecta, rheumatism, multiple myeloma, leukemia, lymphoma, systemic mast cell hyperplasia, prior-related osteoporosis, gastrointestinal or biliary diseases, and adverse reactions to drug treatment (anticonvulsants), were excluded (12, 13).

### Basic data

The demographic characteristics and indicators of this study included age, BMI, education level, type of work, race, menarche, menopause, glutamic oxaloacetic transaminase, alkaline phosphatase, creatinine, blood calcium, blood phosphorus, blood vitamin D, urinary calcium, urinary creatinine, outdoor activity time, daily household chores, low back pain time, leg pain duration, leg cramp time, kyphosis, fracture, hypertension, coronary artery disease, diabetes, rheumatoid arthritis, hyperthyroidism, and asthma.

### Statistical analysis

All data were processed using the Statistical Package for the Social Sciences software, version 17 (SPSS Inc., Chicago, IL, USA). Continuous variables with normal distribution were presented as mean and standard deviation and compared using the Student’s *t*-test. Skewed distributed continuous variables were presented as medians and interquartile ranges and compared using the Mann–Whitney U test. Categorical variables, presented as numbers and percentages of the total, were compared using the Chi-square test. Multivariate logistic analysis was performed for all relevant factors mentioned above to evaluate the risk factors for PMOP. A two-tailed P-value < 0.05 was considered statistically significant.

## Results

The participants had a median age of 60.8 years (range: 44–94 years). Among the 334 postmenopausal women in the study, 35 (10.5%) were diagnosed with osteoporosis. The baseline characteristics of the patients in the OP and non-OP groups are shown in **Table 1**. Univariate analysis of basic data showed that there were statistically significant differences in age, BMI, type of work, alkaline phosphatase, blood calcium, blood calcium, kyphosis, years of smoking, fracture, and asthma between the two groups (P<0.05).

**Table 1:** Characteristic comparison between osteoporosis group and none osteoporosis group females.

Characteristics	Osteoporosis group (n=35)	None Osteoporosis group (n=299)	P
Age (year)^a^	71.0 (66.0, 75.0)	59.0 (53.0, 64.0)	<0.001
BMI^a^	30.6 (28.2,33.3)	36.3 (33.4,40.1)	<0.001
Education level, n (%)			0.197
Illiteracy	15 (42.9)	87 (29.1)	
primary school	13 (37.1)	82 (27.4)	
High school	7 (20.0)	130 (42.5)	
Type of work, n (%)			0.049
physical labor	27 (77.1)	148 (49.5)	
mental labor	8 (22.9)	150 (50.5)	
Race, n (%)			0.750
Han	34 (97.1)	278 (93.0)	
Others	1 (2.9)	18 (7.0)	
Menarche, n (%)	15.0 (14.0,17.0)	15.0 (14.0,16.0)	0.081
Menopause, n (%)	55.0 (46.0,55.0)	50.0 (47.0,51.0)	0.101
Glutamic oxalacetic transaminase (U/L)^a^	18.6 (15.9,25.2)	19.6 (16.5,24.9)	0.373
alkaline phosphatase (U/L)^a^	79.0 (24.8, 110.0)	54.0 (21.3, 84.0)	0.008
Creatinine (umol/L)^a^	64.0 (53.0,72.0)	64.0 (58.0, 77.0)	0.259
blood calcium (umol/L)^a^	2.4 (2.3, 55.0)	2.5 (2.4, 60.0)	0.016
Blood phosphorus (mmol/L)^a^	2.34 (1.57, 3.04)	2.25 (1.30, 2.38)	0.012
vitamin D (g/L)^a^	19.2 (1.3, 24.0)	16.3 (1.2, 24.0)	0.555
Urinary calcium (g/L)^a^	4.18 (2.65, 15.30)	6.33 (2.67, 21.80)	0.099
Urinary creatinine (umol/kg.d)^a^	8150.0 (4.5,11835.0)	4231.0 (2.8,10823.0)	0.107
Total T value^a^	-2.9 (-3.2,-2.6)	-1.0 (-1.6,-0.4)	<0.001
Time spent outdoors (hours)	2.0 (0.0,3.0)	2.0 (0.0,4.0)	0.393
Daily household chores (hours)	2.0 (0.0,4.0)	2.0 (0.0,4.0)	0.811
Low back pain time (hours)	3.0 (0.0,10.0)	2.0 (0.0,6.0)	0.319
Leg pain duration (hours)	2.0 (0.0,10.0)	0.0 (0.0,5.0)	0.156
Leg cramp time (hours)	0.0 (0.0,3.0)	0.0 (0.0,2.0)	0.399
Kyphosis time (years)	0.0 (0.0,0.0)	0.0 (0.0,0.0)	0.006
Fracture, n (%)	5 (14.3)	14 (4.7)	0.020
Hypertension, n (%)	10 (28.6)	71 (23.7)	0.529
Coronary artery disease, n (%)	4 (11.4)	24 (8.0)	0.492
Diabetes, n (%)	2 (5.7)	19 (6.4)	0.883
Rheumatoid arthritis, n (%)	4 (11.4)	27 (9.0)	0.644
Hyperthyroidism, n (%)	1 (2.9)	9 (3.0)	0.960
Asthma, n (%)	2 (5.7)	3 (1.0)	0.030
Years of smoking (years)	0.0 (0.0,0.0)	0.0 (0.0,0.0)	0.015
Daily smoking (branch)	0.0 (0.0,0.0)	0.0 (0.0,0.0)	0.068
Years of drinking (years)	0.0 (0.0,0.0)	0.0 (0.0,0.0)	0.973
Daily drinking (liang)	0.0 (0.0,0.0)	0.0 (0.0,0.0)	0.957

a median (interquartile range).

In addition, logistic multivariate analysis showed that age (odds ratio [OR]: 1.185, 95% confidence interval [CI]: 1.085–1.293, P<0.001) and kyphosis times (OR: 1.468, 95% CI: 1.076–2.001, P=0.015) was positively correlated with postmenopausal osteoporosis, whereas BMI (OR: 0.717, 95% CI: 0.617–0.832, P<0.001), blood calcium (OR: 0.920, 95% CI: 0.854–0.991, P=0.027), vitamin D (OR: 0.787, 95% CI: 0.674–0.918, P=0.002), and outdoor activity time (OR: 0.556, 95% CI: 0.338–0.915, P=0.021) were negatively correlated with postmenopausal OP (**Table 2**).

**Table 2:** Logistic multivariate analysis of different factors and postmenopausal osteoporosis.

Factors	OR	95% confidence interval	P
Age	1.185	1.085-1.293	<0.001
BMI	0.717	0.617-0.832	<0.001
Education level	1.261	0.605-2.626	0.536
Type of work	1.014	0.831-1.237	0.892
Race	1.110	0.473-2.605	0.810
Menarche	1.361	0.998-1.856	0.052
Menopause	1.148	0.997-1.321	0.055
Glutamic oxalacetic transaminase	0.946	0.882-1.014	0.118
alkaline phosphatase	1.004	0.982-1.027	0.730
Creatinine	1.001	0.980-1.022	0.939
blood calcium	0.920	0.854-0.991	0.027
Blood phosphorus	1.754	0.896-3.434	0.101
vitamin D	0.787	0.674-0.918	0.002
Urinary calcium	0.892	0.771-1.032	0.125
Urinary creatinine	1.000	1.000-1.000	0.184
Time spent outdoors	0.556	0.338-0.915	0.021
Daily household chores	1.475	0.951-2.289	0.083
Low back pain time	1.008	0.928-1.095	0.844
Leg pain duration	0.995	0.899-1.102	0.929
Leg cramp time	1.030	0.928-1.143	0.579
Kyphosis	1.468	1.076-2.001	0.015
Fracture	3.196	0.506-20.189	0.217
Hypertension	1.197	0.263-5.454	0.816
Coronary artery disease	0.792	0.137-4.582	0.795
Diabetes	0.978	0.122-7.833	0.983
Rheumatoid arthritis	0.750	0.092-6.125	0.788
Hyperthyroidism	3.552	0.124-101.630	0.459
Asthma	0.479	0.014-15.951	0.681
Years of smoking	0.962	0.878-1.053	0.400
Daily smoking	0.899	0.126-6.434	0.916
Years of drinking	1.015	0.906-1.138	0.794
Daily drinking	0.385	0.074-2.008	0.257

## Discussion

OP and its associated increased risk of fragility fractures are the most disabling consequences of aging in women. Obesity, older age, lower bone density, and 25(OH)D are the main risk factors for hip fractures in postmenopausal women (14, 15); age and fracture history can also predict the risk factors. Moreover, low vitamin D levels also predicted the risk of hip fractures independent of clinical risk factors. The postmenopausal age and fracture incidence are increasing owing to aging populations (16). Hence, interventions to attenuate OP progression and prevent fractures should focus on the older adult population. The present study similarly found a positive association between age (OR: 1.185; 95% CI: 1.085-1.293, P<0.001) and PMOP. In addition, there was a close link between BMI and BMD, confirming the negative relationship between BMD and BMI values; the smaller the BMI, the greater the loss of BMD (15, 17). Another study reported an inverted U-shaped relationship between BMI and lumbar BMD in women and neonates. This finding suggests that increasing BMI may be beneficial for promoting BMD, while an excessively high BMI may be harmful to bone health among women (18). Moreover, BMD values were also associated with BMI in postmenopausal women (19).

The current study found that OP was negatively associated with blood calcium (OR: 0.920, 95% CI: 0.854–0.991, P=0.027) and vitamin D (OR: 0.787, 95% CI: 0.674–0.918, P=0.002) levels in postmenopausal women in Sanya. Dairy products with calcium and vitamin D have a positive effect on bone density in postmenopausal women, and combined supplementation with calcium and vitamin D can prevent osteoporotic hip fractures (20). Plasma calcium plays an important role in the bone remodeling process and can alter the bone structure to meet changing mechanical demands while maintaining bone cell viability and repairing microdamage in the bone matrix (21). Estrogen deficiency leads to many deleterious effects on the bone, including inhibition of osteocyte survival, impairment of osteoblast responses to mechanical stimuli, and repair of aging bone. Reyes-Garcia et al. showed that the daily intake of calcium- and vitamin D-rich milk in healthy postmenopausal women significantly improved vitamin D status, increased femoral neck BMD, and had favorable effects on glucose and lipid profiles (22). Studies have also confirmed that increased serum 25 (OH) D and calcium levels in postmenopausal women can increase skeletal muscle size, strength, balance, and functional task performance while reducing muscle fatigue (23). Furthermore, collagen peptide supplementation enhances the positive effect of calcium and vitamin D supplementation on bone metabolism in postmenopausal women (24).

Menopause is defined as the cessation of menstruation in women, and is associated with ovulatory failure due to oocyte depletion. Menopause is a physiological event that occurs simultaneously with a variety of diseases such as coronary heart disease, stroke, cancer, changes in neuropsychological status and immune function, and bone diseases such as osteopenia and OP. The possible mechanisms underlying the negative association of blood calcium and serum vitamin D levels with OP in postmenopausal women are as follows. First, postmenopausal bone loss may be related to estrogen deficiency and changes in immune status. Postmenopausal OP is a systemic disease characterized by decreased bone mass and increased risk of fractures, mainly due to the significant decrease in estrogen levels after menopause. In addition, the indirect impact of postmenopausal changes in immune status may lead to sustained bone damage, as postmenopausal women typically exhibit a chronic low-grade inflammatory phenotype with changes in both cytokine expression and the immune cell spectrum (25, 26). The second possible mechanism is related to aging. Aging is associated with increased serum parathyroid hormone and alkaline phosphatase levels and decreased serum calcium, phosphorus, and vitamin D metabolites (27). Vitamin D supplementation may improve metabolism in young, postmenopausal, and older adult women. Third, calcium absorption efficiency is an important factor for maintaining calcium balance, which may be related to many cytokines, hormones, growth factors, and reactive oxygen species (24). Fourth, it may be relevant to bone remodeling because of the interference with multiple mechanisms during bone remodeling in the osteoblast and osteoclast lineages. In addition, women with normal bone density and brittle fractures after menopause have lower cortical thickness, and the characteristics of several bone materials in cortical and trabecular mineralized bone tissue are uneven (28).

Patients with postmenopausal OP are prone to serious adverse consequences, such as vertebral compression fractures and thoracolumbar kyphosis (TLK). TLK can cause serious physical, emotional, and economic consequences (29–31). This study found a positive correlation between kyphosis time (OR: 1.468, 95% CI: 1.076–2.001, P=0.015) and postmenopausal OP. A prospective radiological evaluation of the kyphosis index confirmed that high kyphosis is a risk factor for spinal fractures in postmenopausal patients with OP (32). Bernardo et al. also found that older adult women with high kyphosis had a 1.7-fold increased risk of future fractures (33). As the curvature of the chest or waist increases, the local bone density in pre- and postmenopausal women decreases (34). Guo et al. also found that in postmenopausal patients with OP, TLK can occur even without compression fractures and that postmenopausal women are more likely to develop TLK in the future (35).

In addition, outdoor activity duration (OR: 0.556; 95% CI: 0.338–0.915, P=0.021) was associated with PMOP in this study. Age-related bone and muscle losses is associated with physical inactivity and dietary calcium deficiency (36). Studies have suggested that physical activity and muscle strength play a critical role in quality of life, and that physical activity may also reduce the occurrence of sarcopenia (37). Targeted exercise training is the only strategy that can simultaneously improve multiple bone-related and fall-related risk factors. Multiple exercises have a positive impact on bone mass, structure, and strength, and reduce the risk of fractures in postmenopausal women (38). The results of recent studies have emphasized that regular exercise may alleviate postmenopausal symptoms and that even short-term moderate exercise training may significantly reduce these positive effects. Not participating in a supervised high-intensity group exercise program for three months, even with a significant increase in outdoor sports activities, can impact postmenopausal symptoms (39). In addition, research on the influence of high-intensity resistance and impact training (HiRIT) and low-intensity Pilates-based exercise (LiPBE) on the geometric shape of the proximal femur in postmenopausal women has shown that HiRIT can improve the geometric parameters of proximal femoral strength and reduce the risk of hip fracture, whereas LiPBE exercise is largely ineffective (40). Therefore, this implies that the female population with PMOP, particularly the older adults and/or those with severe OP, should be careful when choosing appropriate exercise methods and intensity.

This study is the first to identify risk factors related to fractures and OP in postmenopausal women in Hainan Province, China. As Hainan Province is located in a subtropical region of China, postmenopausal women should make appropriate adjustments based on the strong local sunlight, outdoor work hours, and dietary habits to prevent OP. Epidemiological studies on postmenopausal centenarians and older adult females in Hainan Province have suggested that estrogen is associated with bone resorption and formation (41). Notably, the European postmenopausal guidelines, which state that postmenopausal women should consume 800–1200 mg of calcium and sufficient dietary protein per day. For postmenopausal women with an increased risk of fractures, a daily intake of 800 IU of cholecalciferol is recommended. We further considered supplementing patients with a risk or evidence of vitamin D deficiency, with vitamin D. Regular engagement in weight-bearing exercises based on individual needs and abilities is also recommended (42).

This study has the following limitations. First, it was a cross-sectional study with a limited sample size, and only included participants from a single region. Second, we did not examine the effects of estrogen on postmenopausal OP. Third, a large-sample, multicenter, randomized controlled study is required to confirm the results of this study.

In conclusion, this study found that low BMI, blood calcium and vitamin D levels, kyphosis time, and outdoor activity time were independent risk factors for OP in postmenopausal women. Based on the results of this study, the recommendations for preventing PMOP are as follows. Postmenopausal Chinese women, especially those in Hainan Province, should actively correct hypocalcemia, supplement with vitamin D, and increase their outdoor activity time.

## Data availability statement

The original contributions presented in the study are included in the article/supplementary material. Further inquiries can be directed to the corresponding authors.

## Ethics statement

The studies involving humans were approved by PLA General Hospital of Hainan Hospital. The studies were conducted in accordance with the local legislation and institutional requirements. The participants provided their written informed consent to participate in this study.

## Author contributions

GT, TC, and ZF contributed to the design of the study and the review of the literature. LF, YP, and ZG participated in data collection, analysis and drifting of the manuscript. GT and LF are contribute equally to this work. All authors contributed to the article and approved the submitted version.
